# Indoor positioning using circle expansion-based adaptive trilateration algorithm

**DOI:** 10.1186/s43067-023-00075-4

**Published:** 2023-02-15

**Authors:** Kwame Ibwe, Simeon Pande, Abdi T. Abdalla, Godwin Mruma Gadiel

**Affiliations:** Department of Electronics and Telecommunications Engineering, College of Information and Communication Technologies, University of Dar es Salaam, Dar es Salaam, Tanzania

**Keywords:** Adaptive trilateration, Indoor positioning, Internet-of-Things, RSSI

## Abstract

The increasing availability of mobile devices with wireless communications capabilities has stimulated the growth of indoor positioning services. Indoor positioning is used to locate, in real time, devices’ positions for easy access. The indoor positioning, however, is challenging compared to outdoor positioning due to the large number of obstacles. Global positioning system is ideal for outdoor localization but fails in indoor environments with limited space. Recent development of the Internet of Things (IoT) has brought forth portable and cost-effective wireless technologies that can be used for indoor positioning. In this work, an adaptive trilateration algorithm based on received signal strength indicator (RSSI) was proposed. To assess the positioning accuracy of the proposed algorithm, Bluetooth Low Energy (BLE), Wi-Fi (IEEE 802.11n), ZigBee and LoRaWAN IoT technologies were used. Results show that the error performance is improved by 4% in BLE, 17% in ZigBee, 22% in Wi-Fi and 33% in LoRaWAN when compared to the existing related work.

## Introduction

The demand for precise navigation and positioning systems is becoming stronger and fierce due to ever-emerging applications of location-aware computing. The existing systems like Global National Satellite System (GNSS) that use signals transmitted in the Gigahertz region of the radio-frequency spectrum are highly challenged to provide accurate position estimates due to multipath propagation of signals [1]. The integration of GNSS and Inertial Navigation System (INS) has the potential of combating strong interference. However, the urban indoor environments are becoming increasingly complex, with reflecting and diffracting metal surfaces which make it extra challenging for users to achieve normal high-precision positioning through GNSS–INS indoors [2]. Recent development of machine learning and Internet of Things (IoT) have evolved the robot’s platforms by giving them individual intelligence to self-operate in indoor environments [3]. The new applications of robots are used in warehouses, retail and manufacturing industries for items sorting, delivering and management. Warehouses and industries are filled with repeatable, process-oriented, and error-prone operations. Robotics and automation can take over the repetitive tasks (picking, receiving, putting-away) from humans to achieve more consistent, accurate, and productive warehouse operations [4, 5] . It is therefore important for robots to know where they are in order to perform even more position-efficient tasks. Automated robots can navigate on the warehouse floor freely by avoiding obstacles and walking on the shortest path between two points. They can also be used in hospitals to perform high-risk activities like delivery of medicines during pandemics like COVID-19. Likewise, for obstacle avoidance, ultrasonic sensor modules and preloaded intelligence are used [6]. The radio-frequency identification (RFID) scanner is used to identify and verify destination and recalibrate position of the robot on a path by detecting other locations on the given path [7]. The accuracy of sophisticated satellite and ultrasonic-based positioning systems currently available are limited to line of sight in indoor environments [4]. Therefore, a high-precision adaptive algorithm is desired for complex indoor environments. The desire has increased in recent years due to the developments in machine-to-machine interfaces and IoT. Through IoT, new low-cost gadgets and wearables have been developed. These include Bluetooth Low Energy (BLE) beacons capable of being integrated into robots or devices in warehouses or supermarkets for easy localization. Also, there have been developments of Wi-Fi, ZigBee and LoRaWAN wireless technologies, which can be used for indoor localization with minimal hardware configurations [8]. Therefore, development of machine-to-machine interfaces and IoT form important features of current and future high-accuracy positioning systems [9–11]. The contribution of this paper is twofold: first, improving position accuracy by coupling the trilateration algorithm with the circle expansion stage; second, increasing efficiency by using IoT-based communication technologies to locate objects in indoor environments.

The rest of the paper is organized as follows: The “Related work” section discusses the related literature. “Materials and methods” section details the development of the proposed adaptive trilateration algorithm. “Results and Discussion” section presents and discusses the results relative to the existing literature, and “Conclusion” section concludes the paper.

## Related works

Due to multipath fading in an indoor environment, which is constantly changing, different algorithms have been developed to fit a particular environment. These changes in the indoor environment are inevitable and decrease the overall accuracy of the developed indoor positioning algorithms [12]. Several indoor positioning systems have been developed based on different wireless technologies. Wireless sensor networks (WSNs) using ZigBee technology have been well researched and used for indoor positioning systems using low power, low memory and low computation devices [13–16, 2]. However, there are limited works on adaptive indoor positioning algorithms capable of self-operating robots and industrial automation [17].

A novel 3D adaptive algorithm for WSN was presented in [18]. The algorithm works by initially connecting all non-anchor nodes to anchor nodes and then forming initial smaller groups. At the end, the nodes measure and correct positioning error between the non-anchor node (the missing/disconnected non-anchor node from the group) and anchor node when changes in environment occur. This is successfully done by maintaining neighbor tables between the nodes.

The fading nature of channel propagation for WSN was tested in [19]. The authors proposed a third-order lognormal path-loss model for indoor adaptive positioning algorithm. The algorithm builds and updates the table using received signal strength indicator (RSSI) received in fixed nodes in different environment conditions. In the locating phase, the algorithm chooses the best approximation from the table. The algorithm shows a good relationship between fading channel and node position by giving an average accuracy of 75% compared to other algorithms. Nevertheless, the authors showed that the estimated error close to the wall/obstacle was greater compared to the position in the middle of the room. This is because of multipath on positions close to obstacles. The maximum error achieved was 0.35 m in $10\times 8.8$ square meter area. The position error depends on the room size and positions picked for analysis.

The authors in [20] used received signal strength to estimate the node distances in WSN assuming line of sight (LOS). For a highly changing environment, however, non-existence of LOS could lead to higher positioning error. The authors in [21] presented a localization algorithm based on RSSI ranging scoped which uses fixed parameters in the propagation model to reduce RSSI ranging error. The proposed algorithm, however, creates a one-to-one mapping of RSSI values and distance scope of the parameters and also involves matrix inversions to estimate the unknown coordinates. This renders the algorithm to be complex and less appropriate for highly populated indoor environments. The algorithm uses distance optimization and centroid core triangulation techniques. The algorithm is optimized to fixed obstacles and provides uncertainty to moving obstacles. The authors in [1] proposed an improved robust adaptive algorithm using ultra-wideband (UWB) and microelectromechanical System (MEMS) positioning systems. However, the proposed UWB–MEMS method is easily affected by NLOS errors in indoor environments, which result in low positioning accuracy.

The authors in [11] presented a self-adaptive algorithm using multi-objective optimization for Wi-Fi positioning. The algorithm works by comparing the actual results from the empirical model with the test results by measuring position error. At any particular moment, the algorithm with less position error is used to present overall results. Therefore, the position accuracy is still an open problem because the current measurements are still prone to multipath even though the previous tests ran with good results. In [22], the authors developed a robust trilateration algorithm for indoor positioning systems. The authors used the tags and readers arrangement presented in [20] to obtain position estimates with the assumptions that RSSI pattern distribution is the same in all regions. This technique is adaptive to changes in the indoor environment but prone to errors in the actual estimation of expansion/reduction factor of the intersecting regions. It was shown in [23, 24, 19, 15, 11] that the adaptive algorithms strongly depend on the information stored at training phase (at the offline phase, where by the required pre-information are recorded). This adds memory and power requirements in order to attain accurate position estimation [4, 17, 14].

To address the challenge, this work proposed an adaptive trilateration algorithm using a circle expansion method. The algorithm uses an improved trilateration method and RSSI-distance techniques for positioning and navigation in an indoor factory environment. This work expands on the work of [8] by introducing the adaptive circle-expansion stage in the basic trilateration algorithm. The intention is to increase the accuracy of the distance estimation of the target node using reference nodes in the RSSI-based trilateration algorithm. The IoT wireless technologies like BLE, Wi-Fi, ZigBee and LoRaWAN are used to evaluate the accuracy error performance of the proposed indoor positioning system. This work uses the RSSI data set which is available online.^1^ A comparison of existing adaptive RSSI-based indoor positioning algorithms was performed to obtain the estimation capability in distance error performance. The distance error performance, in meters, was assessed using the PYTHON® simulation platform.

## Materials and methods

### RSSI–distance relationship

**Fig. 1:**
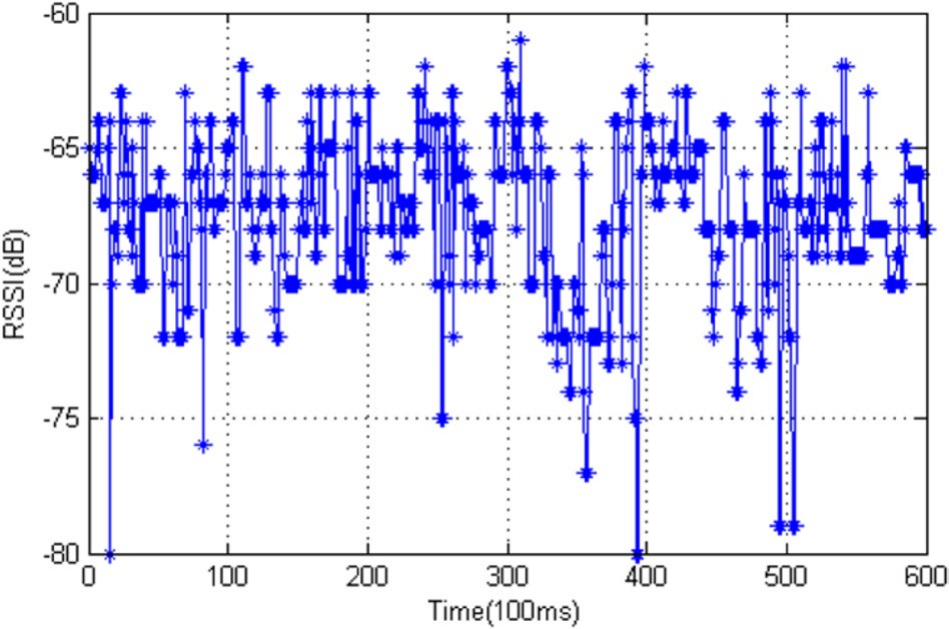
Sample RSSI values in 1 m distance [25]

The RSSI-based positioning is suitable for large-scale applications due to its advantages of low cost and high accuracy. However, it suffers from low stability because RSSI is easily blocked and easily interfered with objects and environmental effects. The RSSI is usually unstable even in a well-controlled indoor scenario due to multipath fading. Authors in [25] conducted an experiment on RSSI–distance relationship, and the results are shown in Fig. 1. The experiment was set with the beacon (sender) being 1m away and facing directly to the mobile device (receiver). The results show that RSSI varies vigorously from -80dB to -61dB. Hence, the authors had to remove the outliers of the RSSI before processing. This work adopts the single direction outlier removal technique used in [25]. The single direction outlier removal is applied to the RSSI, for the reason that RSSI tends to decline due to indoor multipath fading. The outliers of RSSI need to be removed before any further process. Nevertheless, authors in [26] conducted an experiment using Wi-Fi sniffer and found that strong signal had higher confidence than weaker ones in target position estimation, and hence, removing outliers becomes a straightforward exercise.

The proposed adaptive algorithm uses the RSSI distribution characteristics in finding the best distances for position estimation. This is done by sorting and choosing the largest RSSI values for distance estimation. The log-normal indoor propagation model is used to model the indoor propagation based on RSSI as in [27]. This model is represented as (1):

1 $$P_{RX} [dB] = PL(d_{0} ) - 10.\eta .\log_{10} \frac{d}{{d_{0} }} + X_{\sigma } ,\,\,\,\,\,\,\,\,\,\,d > d_{0}$$

where PL(d_0_) is the path loss value for a reference distance *d*_*0*_, *η* is the path loss exponent, and X_σ_ is a Gaussian random variable with zero mean and variance, ${\sigma }^{2}$, that models the random variation of the RSSI value.

### Proposed adaptive trilateration algorithm

Trilateration algorithm uses RSSI measurements to estimate the distance between the tag (targeted node) and reader (reference node) [28]. The distances between reference locations and the target location can be considered as the radii of many circles with centers at every reference location. Hence, the target location is the intersection of all the sphere surfaces. This work adopts the tags’ distance relations derived in [22].

**Fig. 2:**
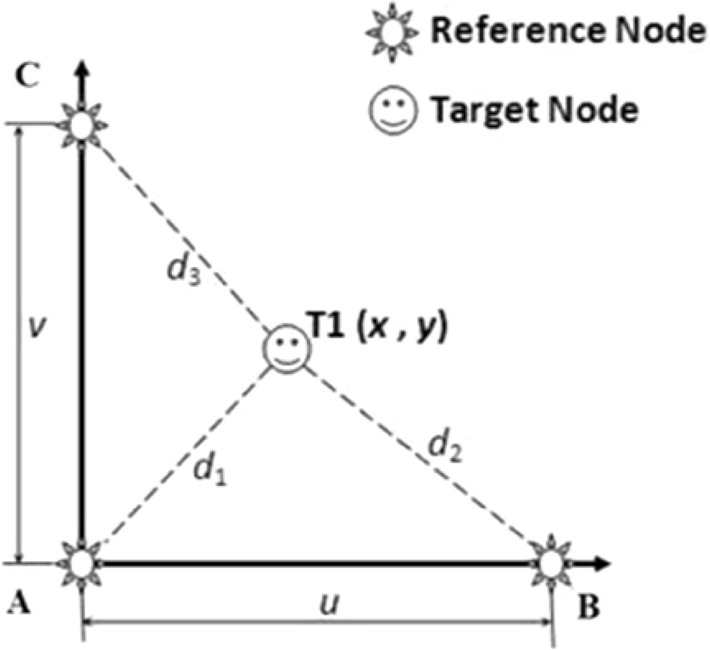
Tags and readers arrangement [22]

Figure 2, adopted from the work of [22], describes the arrangement of the reference nodes (A, B and C) and targeted node (T1) in a simplified fashion. The reference sensor nodes are located at the corners of the triangular area. This method only requires three reference nodes for trilateration. Node A(*x*_*1*_*,y*_*1*_) and B(*x*_*2*_*,y*_*2*_) are used to get the $x$ value, while C(*x*_*3*_*,y*_*3*_) and A(*x*_*1*_*,y*_*1*_) are used to get the $y$ value; hence, $(x, y)$. The distances among sensor nodes/readers (*d*_*1*_*, d*_*2*_ and *d*_*3*_) are obtained using a log-distance path loss model to convert RSSI values to distances from the previous process.

Adopting the values $x_{1} = 0,x_{2} = u,x_{3} = 0,y_{1} = 0,y_{2} = 0,y_{3} = v$ gives:

2 $$x = \frac{{u^{2} + \left( {d_{1}^{2} - d_{2}^{2} } \right)}}{2u}$$

3 $$y = \frac{{v^{2} + \left( {d_{1}^{2} - d_{3}^{2} } \right)}}{2v}$$

**Fig. 3:**
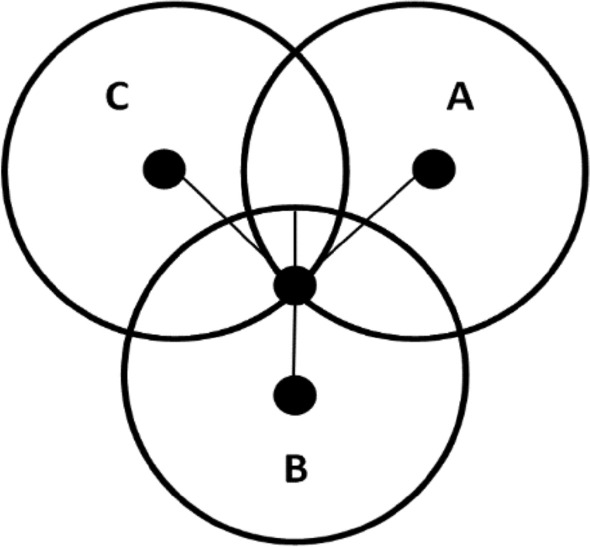
Intersection of two circles A and B [20]

**Fig. 4:**
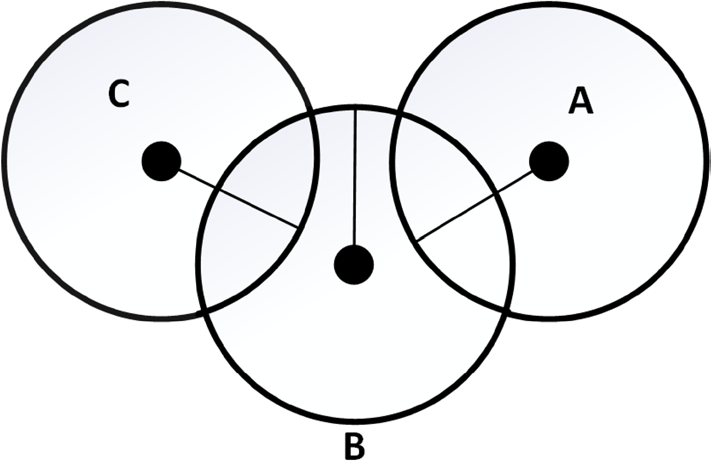
No common intersection [20]

The received signal power is affected by propagation loss and is sensitive to channel interference, attenuation, reflection, fading and shadowing [20]. The position of the tag is prone to error in this way because the intersection point is affected by the RSS value. Due to the interference, multipath and noise, the three circles may not intersect with a common point as shown in Fig. 3. The two circles A and B are intersecting at a point but circle C is not. The same can be seen in Fig. 4 where all three circles are not intersecting at a common point either, so in that case increasing the number of anchors could give better results.

**Fig. 5:**
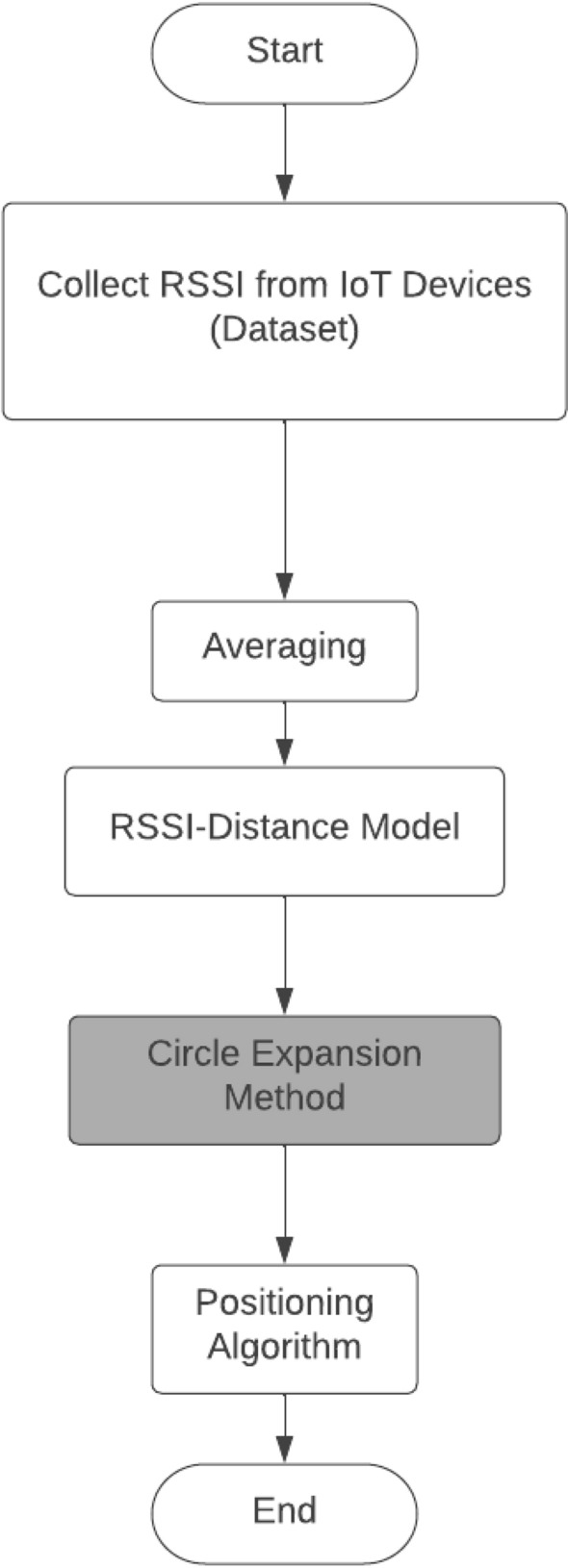
Proposed adaptive positioning algorithm

**Fig. 6:**
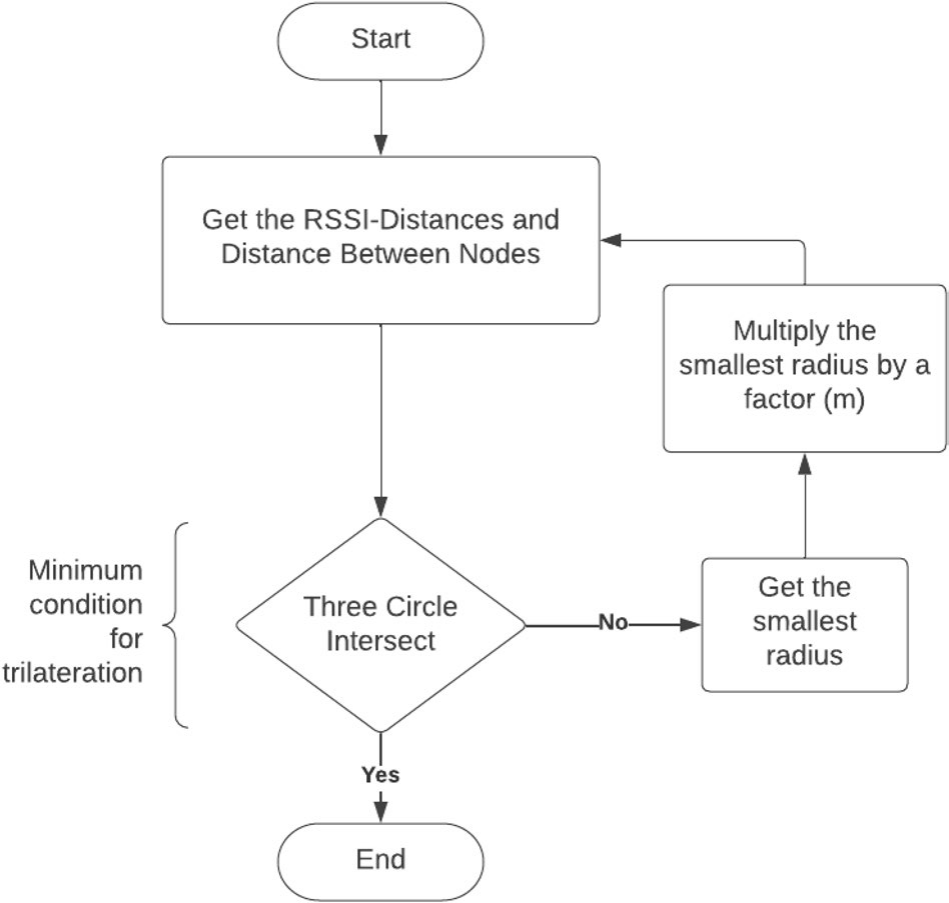
Circle expansion method

To ensure that the three circles always intersect at a common point for accurate readings, this work proposes an adaptive algorithm which makes use of geometry theories for accurate positioning estimation (Fig. 5). The algorithm includes a circle expansion method to check whether the three circles intersect (Fig. 6). Figure 6 summarizes flow of activities in the circle expansion algorithm. It is assumed that the RSSI distribution is the same on all tags/nodes regions.

To check whether three circles intersect normal circles properties are used. If the distance obtained from RSSI measurement is *d*_*1*_*, d*_*2*_* and d*_*3*_ for circles 1, 2 and 3, respectively, then the following rules are used to check whether there is an intersection as described in Fig. 6.

4 $$\begin{gathered} r_{1} + r_{2} > d_{1} \hfill \\ r_{3} + r_{2} > d_{2} \hfill \\ r_{1} + r_{3} > d_{3} \hfill \\ \end{gathered}$$

**Fig. 7:**
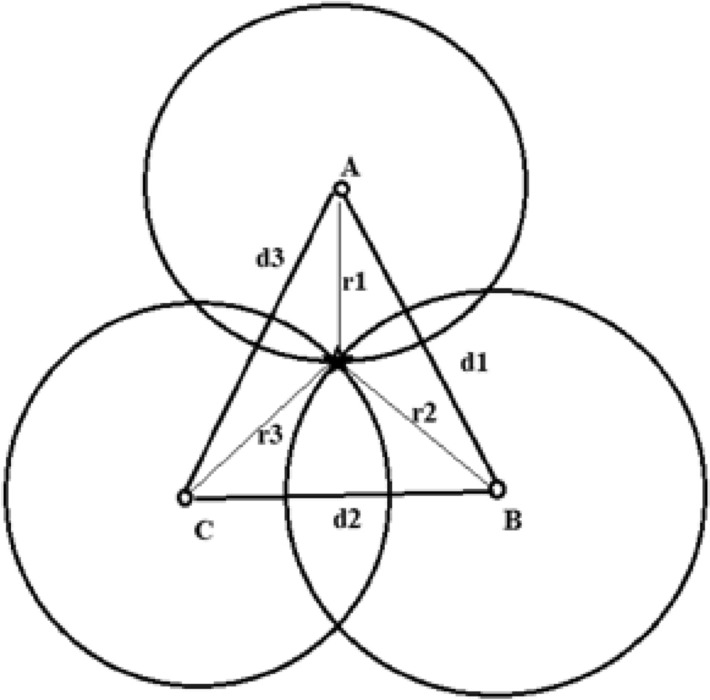
The reduced intersection point of the circles A, B and C

If two circles intersect, then the distance between their center points should be smaller than the sum of their respective radii. If there is no intersection, the radius of the smallest RSSI-distance is increased by factor $m$, then the trilateration algorithm is performed again to get the position. The process is repeated until the three circles give accurate estimates. Hence, the target location is the intersection of all the sphere surfaces as shown in Fig. 7.

The value of $m$ is adjusted as described in the work of [22]. It is always positive and is estimated as the minimum value between the three equations as shown in (5).

5 $$m = \min \left\{ \begin{gathered} d_{1} - r_{1} - r_{2} \hfill \\ d_{2} - r_{2} - r_{3} \hfill \\ d_{3} - r_{2} - r_{1} \hfill \\ \end{gathered} \right\}$$

### Environmental modeling

To evaluate the performance of each wireless technology, two environments were built as described in [8]. The first environment was selected to be a typical research laboratory with dimensions $10.8 m \times 7.3 m$. The environment was selected due to the large size with large numbers of equipment, computers, Wi-Fi and BLE devices that could impose interference, mimicking a noisy environment for experimenting. The second selected environment had dimensions of $5.6 m \times 5.9 m$ representing a small meeting room. The second environment was a perfect testing area as it demonstrated conditions contrasting those in the first environment. The second environment had much smaller space that contained only tables and chairs. No equipment, devices or computers were present in the environment that could cause significant interference in the area, creating a low-noise environment for testing. The parameters used for environment 1 and environment 2 are shown in Tables 1 and 2, respectively.

**Table 1:** Parameters used in environment 1

	Wi-Fi	BLE	ZigBee	LoRaWAN
$\eta$	2.013	2.511	2.261	1.246
$X_{\sigma }$	− 49.990	− 75.540	− 51.100	− 31.380

**Table 2:** Parameters used in environment 2

	Wi-Fi	BLE	ZigBee	LoRaWAN
$\eta$	2.162	2.271	1.653	0.519
$X_{\sigma }$	− 45.730	− 75.480	− 51.010	− 33.440

**Fig. 8:**
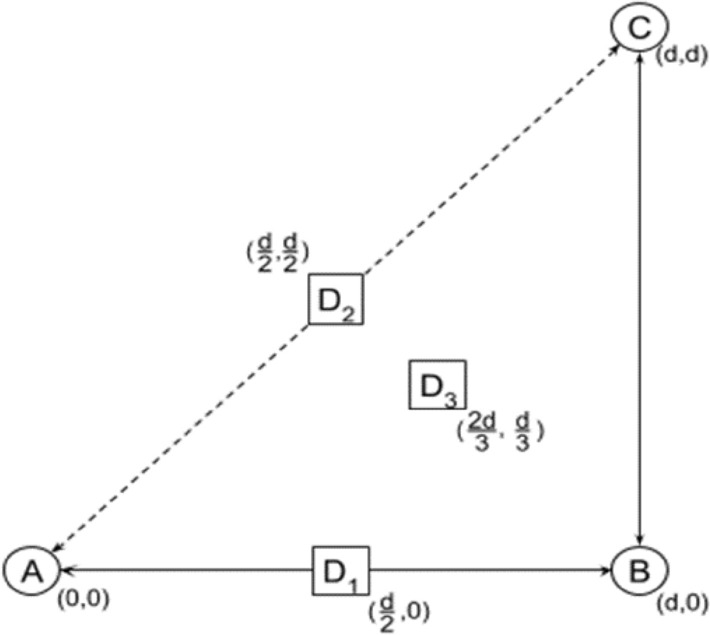
Experimental setup [8]

To set up for the experiments of the two environments, the arrangement in Fig. 8 was set up. The right-angle triangle was created between the nodes. The distances of the triangle, *d*, between nodes A, B and C were set to be equal. The experiments used three selected distances for testing at 1, 3 and 5 m. The receiver was set to one of three positions: in the center between nodes A and B (*D*1), in the center between nodes A and C (*D*2) and in the centroid of the triangle (*D*3). The target locations are given in Table 3. The three distances were tested using the different wireless technologies, Wi-Fi, BLE, ZigBee and LoRaWAN, while keeping the same arrangement and adjusted target positions *D1, D2 and D3.*

**Table 3:** Targets location at given distances

Location (m)	Test points coordinates		
	D1 (m)	D2 (m)	D3 (m)
1	(1/2,0)	(1/2, 1/2)	(2/3, 1/3)
3	(3/2,0)	(3/2, 3/2)	(2, 1)
5	(5/2,0)	(5/2, 5/2)	(10/3, 5/3)

The theoretical propagation models were developed and simulated in PYTHON using (1) and the parameters given in Tables 1 and 2. The channel models were developed for each wireless technology using the publicly available RSSI dataset.^2^ Nine tests were done for each wireless technology based on varying the distances in Table 3. In each of the tests, the location of all the nodes was recorded along with the measured RSSI values. The measured RSSI values were used to approximate the position of the receiver with respect to reference nodes.

To evaluate the accuracy of the wireless technologies used, the mean-squared error (MSE) of the actual and approximate distance was used. The MSE is measured as:

6 $$\text{MSE}=\frac{1}{n}{\sum }_{1}^{n}\sqrt{{\left(x-{x}_{i}\right)}^{2}+{\left(y-{y}_{i}\right)}^{2}}$$

where $n$ is the number of nodes. $x$ and $y$ are the actual and *x*_*i*_ and *y*_*i*_ are the estimated coordinates of the target node.

The results were then passed to Microsoft Excel for further data analysis. The MSE in each environment were estimated and analyzed. To demonstrate adaptation, the developed algorithm has to show smaller MSE compared to the basic RSSI algorithm with respect to environment changes.

## Results and discussion

The accuracy evaluation between the wireless technologies, based on minimum MSE given in (6), was performed. The results for environment 1 and environment 2 are shown in Tables 4 and 5, respectively. Likewise, Table 6 presents the overall error performance for each environment.

**Table 4:** MSE values with distances in environment 1

Distance(m)	Test point	Actual coordinates (m)		Error (m)			
		M	N	BLE	Wi-Fi	LoRaWAN	ZigBee
1	D1	0.5000	0.0000	0.1321	0.1325	0.3250	0.3390
	D2	0.5000	0.5000	0.0005	0.0005	0.2955	0.3312
	D3	0.6667	0.3333	0.1792	0.9316	0.4535	1.7652
	**Average**			**0.1039**	**0.3548**	**0.3580**	**0.8118**
3	D1	1.5000	0.0000	1.4993	2.0832	0.1294	0.8879
	D2	1.5000	1.5000	0.0019	0.0018	0.0011	0.7184
	D3	2.0000	1.0000	0.7056	0.4511	0.7356	0.5024
	**Average**			**0.7356**	**0.8453**	**0.2887**	**0.7029**
5	D1	2.5000	0.0000	2.3983	1.3020	1.6235	0.1399
	D2	2.5000	2.5000	0.0024	0.0011	0.3130	0.0018
	D3	3.3333	1.6667	1.0766	0.9561	1.1772	1.3564
	**Average**			**1.1591**	**0.7530**	**1.0379**	**0.4993**

**Table 5:** MSE values with distances in environment 2

Distance(m)	Test point	Actual coordinates (m)		Error (m)			
		M	N	BLE	Wi-Fi	LoRaWAN	ZigBee
1	D1	0.5000	0.0000	0.4998	0.1420	0.5000	0.4998
	D2	0.5000	0.5000	0.7593	0.0131	0.0004	0.0501
	D3	0.6667	0.3333	0.2357	0.0657	0.2358	0.2355
	**Average**			**0.4983**	**0.0736**	**0.2454**	**0.2618**
3	D1	1.5000	0.0000	1.4998	0.2455	1.4990	1.3829
	D2	1.5000	1.5000	0.0019	0.4813	0.0002	0.0010
	D3	2.0000	1.0000	0.7067	0.9992	2.6789	0.9304
	**Average**			**0.7361**	**0.5753**	**1.3927**	**0.7714**
5	D1	2.5000	0.0000	2.2334	0.4965	2.4986	1.1639
	D2	2.5000	2.5000	0.0016	0.2235	0.0018	1.5782
	D3	3.3333	1.6667	1.0160	0.7874	1.8097	1.4564
	**Average**			**1.0836**	**0.5024**	**1.4367**	**1.3995**

**Table 6:** Average position error (m)

Wireless technology	Environment 1	Environment 2	Overall
BLE	0.666	0.773	0.719
Wi-Fi	0.651	0.384	0.517
LoRaWAN	0.562	1.025	0.793
ZigBee	0.671	0.811	0.741

In Environment 1, the BLE produced an error of 0.666 meters while in Environment 2 it was 0.773 meters. Using the proposed adaptive circle expansion method, LoRaWAN has demonstrated to have the best error performance in Environment 1 with 0.562 meters. However, its overall performance of 0.793 meters is the worst of the four technologies. Wi-Fi has demonstrated to be the second-best technology in Environment 1 with 0.651 meters and the best technology in Environment 2 with 0.384 meters. The overall performance of Wi-Fi still places it to the first place with 0.517 meters. ZigBee is the least performing in Environment 1 with 0.671 meters and third in Environment 2 with 0.811 meters. It is also demonstrated to be the third best in overall performance with 0.741 meters.

**Fig. 9:**
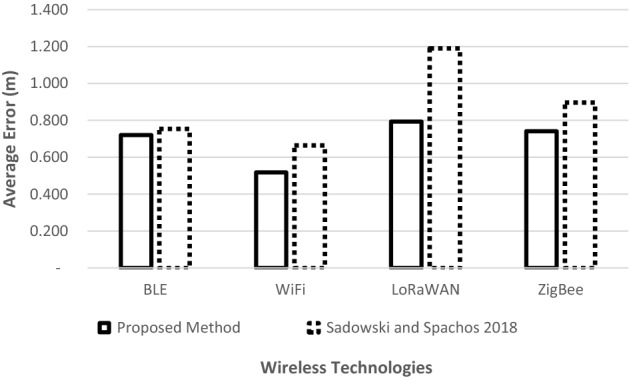
Comparison of the average position error

Environment 1 has a better advantage to LoRaWAN technology whose signals could travel farther distances with less obstructions, reflections and diffractions. In Environment 2, the LoRaWAN deteriorates due to an increased number of objects in the room. It is also observed that Wi-Fi has the best performance in both environments at all distances of 1, 3 and 5 meters at test points D2, because of lower amount of interference. However, at the edges of the triangle as shown in Fig. 8, the devices experienced high interference levels hence degrading the estimation accuracy. Figure 9 compares the overall performance of the proposed method with the existing work done by (Sadowski and Spacho, 2018). It is observed that the proposed method outperforms the existing method, in terms of the estimation accuracy, by 4% in BLE, 17% in ZigBee, 22% in Wi-Fi, and 33% in LoRaWAN.

In this study, the inclusion of the adaptation stage in the trilateration algorithm has revealed useful insights. Overall, it is observed that error performance improves for Wi-Fi and BLE wireless technologies. The Wi-Fi produces the best estimates with an average error of 0.517 meters. However, the Wi-Fi devices use main power which brings the challenge of power consumption for battery-powered devices. On the other hand, BLE has the second highest accuracy with an average error of 0.719 meters. The BLE beacon is the system that also consumes the least amount of power, but it has the lowest transmission range for all the tested devices. In addition, due to the low current draw of BLE, rechargeable batteries could be used in order to power the device, which could reduce the overall system’s cost. The major disadvantage of using BLE is that it would not be suitable for covering a large area due to its poor transmission range, and therefore, additional devices would be required. It is also observed that LoRaWAN could be a cost-effective technology due to long-range capability needing fewer nodes. However, its high-power consumption brings a challenge compared to BLE technology. The experimental results confirm that despite the better average error performance of Wi-Fi technology, BLE could be the best choice due to its portability and battery powering ability. The Wi-Fi and LoRaWAN, however, are ideal for medium and longer ranges, respectively.

## Conclusion

It has been shown that modeling an indoor environment is challenging, especially in the presence of walls, furniture, electronic devices and movement of people and objects in small confined spaces. This forces indoor positioning systems to be specific for a given environment and hence lacks proper standards. This research work presents an adaptive trilateration algorithm for RSSI-based indoor positioning systems. The proposed algorithm adapts the changes in different environments by adjusting the estimated distances of the intersecting circles representing the signal coverage of the communicating devices. IoT communication technologies including Wi-Fi, BLE, ZigBee and LoRaWAN were used to provide interconnections with each other and form WSN, which could facilitate the indoor positioning process. Results show that accuracy was improved by 4% in BLE, 17% in ZigBee, 22% in Wi-Fi, and 33% in LoRaWAN, compared to the existing related literature. These results have shown that improved position accuracy could be obtained if the trilateration is coupled with the adaptive circle expansion stage. Likewise, the results have given further insights on the selection of the indoor positioning algorithms.

## Data Availability

All data generated or analyzed in this study are included in the manuscript.

## Footnotes

1 https://github.com/pspachos/RSSIDataset.

2 https://github.com/pspachos/RSSIDataset.
